# Analysis of carbon fiber dust exposure status and related occupational health risk factors among workers during the molding process of carbon fiber products

**DOI:** 10.3389/fpubh.2026.1926373

**Published:** 2026-09-04

**Authors:** Andong Hu, Xiaojuan Zhong, Yi Rong, Yuanliang Wu, Cheng Liu, He Cao

**Affiliations:** Shenzhen Longhua District Center for Disease Control and Prevention (Shenzhen Longhua District Health Supervision Institute), Shenzhen, Guangdong, China

**Keywords:** carbon fiber products, dust, occupational exposure, occupational health, risk factors

## Abstract

**Objective:**

To analyze the exposure of workers to carbon fiber dust during the manufacturing process of carbon fiber products, and to assess the occupational health risk level and related risk factors among the exposed population.

**Methods:**

A total of 955 workers engaged in carbon fiber product molding positions from 30 carbon fiber manufacturing enterprises in Shenzhen were selected. All workers underwent testing for carbon fiber dust exposure. The exposure status of hazardous factors among workers exposed to carbon fiber dust was analyzed, and questionnaire surveys and scale assessments were conducted. The quantitative assessment results of the International Council on Mining and Metals (ICMM) occupational health risk assessment model for workers exposed to carbon fiber dust were analyzed, and groups were divided according to their ICMM occupational health risk levels. Variables were screened via LASSO regression, followed by the construction of a binary Logistic regression model to analyze risk factors associated with occupational health risks among workers exposed to carbon fiber dust.

**Results:**

Carbon fiber dust exposure was identified in 8.38% of employees, and the number of exposed employees varied significantly across different posts (P < 0.05). Per the ICMM occupational health risk assessment for workstations with carbon fiber dust-exposed employees, there were 3 cases of tolerable occupational health risk, 36 potential risk cases, 37 high-risk cases, 2 very high-risk cases, and 2 intolerable risk cases. A total of 5 variables were screened out by LASSO regression, regression analysis revealed that risk factors contributing to elevated occupational health risks among carbon fiber dust-exposed employees included years of prior frontline manufacturing work (OR = 5.018, 95% CI = 2.728–9.231) and employment in grinding workshops (OR = 3.111, 95% CI = 1.123–8.624). Protective factors against such risks encompassed high awareness of occupational disease prevention (OR = 0.739, 95% CI = 0.595–0.919) and college or bachelor's educational attainment (OR = 0.131, 95% CI = 0.024–0.699).

**Conclusion:**

Workers engaged in carbon fiber product molding face a certain risk of carbon fiber dust exposure. The occupational health risks of employees exposed to carbon fiber dust are associated with years of prior frontline work, educational background, personal protective behaviors, and other relevant factors.

## 1 Introduction

With the continuous iteration of new materials worldwide, carbon fiber products occupy an indispensable position in high-end manufacturing and related emerging fields owing to their superior multi-dimensional properties. Carbonized and graphitized organic fibers yield graphite materials with microcrystalline structures. These graphite microcrystals are lamellar, and repeated stacking of them along the fiber axis produces usable carbon fiber materials. This material boasts profound application value in aerospace, automobiles, industrial robots, precision instruments, sports and leisure goods and other sectors. In 2025, China's Ministry of Human Resources and Social Security added 17 new occupations including carbon fiber molding workers (1). This policy provides clear guidelines for career development, job promotion and standardized management of practitioners in relevant industries, making standardized administration of corresponding posts an imperative.

Occupational protection is a core concern for all industries with special working environments. Carbon fiber product manufacturing consists of two key procedures: prepreg production and molding processing, and carbon fiber molding workers are mainly engaged in molding operations. Operators may be exposed to various occupational hazards at every stage of actual production (2, 3). Among these hazards, carbon fiber dust generated during carbon fiber machining constitutes a major occupational exposure for workers in this occupation. Exposure to carbon fiber dust mainly damages the respiratory system, skin and eyes, causing multi-system adverse health effects. An occupational exposure risk study on insulator workers exposed to multiple materials (4) confirmed a significant correlation between carbon fiber dust exposure and recurrent thoracic infections. Virgin carbon fibers have tiny diameters and lengths of only several millimeters; they break into finer fragments during processing. These fibrous residues maintain high carbon content, which endows carbon fiber dust with prominent chemical inertness and strong biopersistence, making it difficult for the human immune system to degrade and eliminate after entering the body (5). In addition, thermally treated carbon fiber fragments induce more severe inflammatory responses and genotoxicity than virgin carbon fibers (6), raising the incidence of relevant occupational diseases. The processing of certain carbon nanotube-reinforced carbon fiber composites produces not only carbon fiber dust but also carbon nanotubes. Scholars have issued warnings (7) that inhaled carbon nanotubes pose health risks similar to asbestos, and they compared the two substances from four perspectives: acicular morphology, biopersistence, high specific surface area, and strong capacity for surface adsorption of toxic substances. Although carbon fiber molding workers are primarily exposed to polyacrylonitrile (PAN)-based carbon fibers in daily work, potential health risks posed by additives such as carbon nanotubes cannot be ignored.

At present, domestic literature concerning the occupational health status of carbon fiber molding workers is scarce, and no published studies have explored influencing factors of carbon fiber dust exposure. Against the backdrop of growing attention to the health and safety of workers in emerging occupations, this study first identified employees with carbon fiber dust exposure. It then adopted the occupational health risk assessment model developed by the International Council on Mining & Metals (ICMM) to analyze occupational health risks among this population and further evaluate relevant occupational risk factors. The full research content is presented as follows. 2 Methods 2.1 Subjects and Methods Thirty enterprises engaged in carbon fiber product manufacturing in Shenzhen were selected for this study, including 10 sports goods companies, 2 wind energy equipment companies, 4 automotive equipment companies, 4 unmanned aerial vehicle companies, 4 aerospace enterprises, and 6 other carbon fiber product manufacturers. All these enterprises use carbon fiber prepregs as their main raw material. The core responsibilities of carbon fiber product forming technicians involve prepreg molding and processing, including vacuum infusion, compression molding, filament winding, hot-press curing, rolling, layering, cutting, wrapping, engraving, grinding, flat lapping, preforming, forming, and packaging. A total of 955 frontline production workers were randomly selected to participate in the study. Inclusion criteria were as follows: (1) Workers employed in core positions related to carbon fiber product forming; (2) Employment duration in the current position exceeding 3 months; (3) Voluntary informed consent obtained from all participants. Exclusion criteria were as follows: (1) Pre-existing occupational pneumoconiosis, obstructive pulmonary disease, pulmonary fibrosis, or other severe systemic diseases prior to employment in the current position; (2) Presence of cognitive impairment; (3) Actual on-the-job working time less than two-thirds of the required working hours for the position. This study was approved by the Biomedical Ethics Committee of Shenzhen Longhua District Chronic Disease Prevention and Control Center (Mental Health Center), entrusted by Shenzhen Longhua District Center for Disease Control and Prevention (Shenzhen Longhua District Health Supervision Institute). All participants provided written informed consent. All procedures were conducted in accordance with the 1964 Declaration of Helsinki and its later amendments.

## 2 Methods

### 2.1 Occupational health status survey

A field survey was conducted among the factory and enrolled employees from November 1, 2025 to March 30, 2026. Factory-related investigation indicators mainly included the layout of carbon fiber product molding workshops, noise decibel value, ambient temperature, personal protective measures, and occupational health management. Paper-based questionnaires were used to collect individual information covering gender, age, educational attainment, working years, job position, personal medical history, and protection capability. The evaluation of personal protective behaviors was based on the national standard series Specification for the Allocation of Personal Protective Equipment (GB 39800) (8), and corresponding standard modules were selected according to different job characteristics. A self-designed Questionnaire on Occupational Hazard Cognition and Self-Protection (hereinafter referred to as the Occupational Protection Awareness Questionnaire) was adopted in this study. The questionnaire was evaluated and revised by occupational health professionals including senior manufacturing practitioners and health administrative staff. The Cronbach's α coefficient of the scale was 0.85. The total score of the questionnaire ranged from 0 to 90 points, and a higher score represented a higher level of occupational protection cognition.

### 2.2 Judgment criteria for carbon fiber dust exposure

This study adopted *Occupational Exposure Limits for Hazardous Factors in the Workplace – Part 1: Chemical Hazardous Factors* (GBZ 2.1-2019) (9) as the evaluation basis. Dust sampling and detection were carried out in strict accordance with the requirements of GBZ 159 (10) and GBZ/T 192 (11). An SP-30s dual-channel dust sampler manufactured by Suzhou Lanhua Instrument Co., Ltd. was used, equipped with 40 mm-diameter perchlorovinyl filter membranes and matched dust sampling clamps, with the sampling flow rate set at 20 L/min. The sampling pump was calibrated before and after sampling at the calibration point of 20 L/min. Each calibration was repeated three times, and the average value was adopted. The quality control requirements were consistent with those for flue gas condensed particulate matter. The relative deviation between the calibrated flow rate and the set flow rate was controlled within ±5%, and the relative deviation between pre-sampling and post-sampling calibration values was no more than 5%. Samples were deemed invalid if the deviation exceeded the above range (12). Monitoring points were arranged at operating posts in the workplace, including dust-generating points in the workshop, regular working areas of employees, and breathing zone areas with a fixed breathing height of 1.5 m. The sampling duration was determined according to the actual working hours of each post. Continuous 8-h sampling was performed for employees with a daily working duration of more than 8 h, and the obtained result was defined as the 8-h time-weighted average concentration (CTWA). For employees with a daily working duration of less than 8 h, continuous sampling was conducted for 15 min per day for three consecutive days, and the average value was calculated. The working-hour weighting method was applied for subsequent concentration calculation, as shown in Equation (1):


$$\begin{array}{l} C_{TWA}=\frac{C_{15\min}\times T_{work}\times C_{background}\times T_{non-work}}{8}, \end{array}$$ (1)

This formula is used to convert the measured concentration obtained from a 15-min sampling test into the 8-h Time-Weighted Average concentration (CTWA). Twork refers to the total daily working duration of the employee at the corresponding post during sampling; Cbackground represents the exposure concentration of carbon fiber dust to which the employee is exposed during non-working periods (which may be set to 0 for calculation purposes).

Field blank samples shall be prepared for each sampling scenario synchronously. The field blank filters adopt dust-measuring filter membranes from the same batch and serial number segment as the test samples. All operational procedures are identical to those for test samples, except that the blank membranes are not connected to a sampling pump and no air is drawn through them. Upon completion of laboratory analysis, the absolute mass gain of each field blank filter membrane shall be less than 0.1 mg. All samplers involved shall be qualified professional technical staff formally registered with the occupational health technical service institution.

For carbon fiber dust (inhalable total dust) in the workplace, the 8-h Permissible Concentration-Time Weighted Average (PC-TWA) shall not exceed 3 mg/m^3^.

To strengthen the control over carbon fiber dust exposure, the management concentration requirements specified in GBZ/T 224-2010 *Terminology for Occupational Health* shall be adopted as supplementary control thresholds: (1) Where the calculated CTWA≥1.5 mg/m3, occupational exposure is deemed to exist, and corresponding occupational exposure management measures shall be initiated; (2) Where the calculated CTWA exceeds the PC-TWA limit, the exposure to carbon fiber dust shall be judged as over-limit exposure.

### 2.3 Health risk assessment criteria

The ICMM occupational health risk assessment model (13) was adopted in this study, which includes quantitative assessment after assignment and qualitative assessment by matrix method. This study adopted the former. The quantitative formula was RR = C × PrE × PeE × U, with the letters on the right side of the equation being assignable. Detailed content is shown in Table 1. Regarding the scoring criteria for each item in this model: For the C value, the criteria were based on the original ICMM text: 1 point: Extremely low exposure level, no adverse health effects; 15 points: Can cause reversible, non-fatal health reactions, such as upper respiratory tract irritation, eye irritation, skin irritation; 50 points: Can cause permanent but non-life-threatening health damage (e.g., mild lung function impairment); 100 points: Can cause severe permanent injury, disability, or death (e.g., pneumoconiosis, tumor). The scoring criteria for PrE are formulated in accordance with the carbon fiber dust (total dust) occupational exposure limit specified in *GBZ 2.1-2019* (9), where the permissible concentration-time weighted average (PC-TWA) is ≤ 3 mg/m^3^. The evaluation is determined based on the possibility that workers' actual exposure concentration exceeds the Occupational Exposure Limit (OEL): 3 points (Low probability): Time-weighted average concentration (CTWA) < 50% OEL (< 1.5 mg/m^3^), with almost no risk of exceeding the limit; 6 points (Medium probability): CTWA = 50%~100% OEL (1.5~3.0 mg/m^3^), with a potential risk of exceeding the limit; 10 points (High probability): CTWA > 100% OEL (>3.0 mg/m^3^), with confirmed limit excess and an extremely high risk.

**Table 1:** ICMM quantitative assessment details.

Item	Meaning	Assignment/Valuation
C	Health consequences	1 point: No impact on health; 15 points: Health impact but reversible and non-fatal; 50 points: Mild, permanent functional impairment; Maximum 100 points: Severe permanent damage or significantly reduced life expectancy
PrE	Exposure probability	Likelihood of exposure to the hazard: 3 points: Low risk; 6 points: Moderate risk; 10 points: High risk
PeE	Exposure time	Duration of contact with the at-risk position: 0.5 points: Once per year; 1 point: Several times per year; 2 points: Several times per month; 6 points: 2–4 h per shift; 10 points: 8 h per shift
U	Uncertainty factor	Uncertainty of this assessment: 1 point: Certain; 2 points: Uncertain; 3 points: Very uncertain
RR	Risk level	< 20 points: Tolerable risk; 20–69 points: Potential risk of occupational health damage; 70–199 points: High risk of occupational health damage; 200–399 points: Very high risk of occupational health damage; ≥400 points: Extremely high and intolerable occupational health risk

The PeE values are consistent with those listed in Table 1.

The U value is defined as follows: 1 point (Certain): Complete detection data, clear hazard identification, and no controversial findings; 2 points (Uncertain): Partial missing data and moderate hazard evidence; 3 points (Highly uncertain): Severe insufficient data and inadequate hazard evidence.

### 2.4 Observation indicators

(1) The carbon fiber dust exposure status of employees was statistically analyzed. The daily working hours and carbon fiber dust test results (i.e., CTWA values) of exposed employees were collected, and the ratio of detected concentration to the Occupational Exposure Limit (OEL) was calculated, with the detected concentration defined as the CTWA value.

(2) The ICMM quantitative risk assessment results of employees exposed to carbon fiber dust were systematically analyzed.

(3) Based on the ICMM quantitative evaluation results, the occupational health exposure risks of employees were graded, and the influencing factors of occupational health risk were further analyzed.

### 2.5 Statistical analysis

Excel and SPSS 26.0 were used for data sorting and analysis. If the missing data proportion of an employee or an indicator exceeded 30%, all information of that employee or the corresponding indicator would be eliminated; otherwise, multiple imputation was applied to handle missing values. For measurement data, the Kolmogorov-Smirnov (K-S) test and Q-Q plots were firstly adopted to test the normality of data, followed by homogeneity of variance test. Measurement data with normal distribution or insignificant skewness were expressed as mean ± standard deviation and analyzed by the t-test. Skewed data were expressed as median (interquartile range) M(P25,P75) and analyzed by the Mann-Whitney *U* non-parametric test. Count data were expressed as case number (n) and compared via the χ^2^ test. LASSO regression was used for variable screening, binary Logistic regression models were applied to assess influencing factors, and GraphPad Prism 9.5 was adopted for graph plotting. A *P*-value less than 0.05 was defined as the threshold for statistical significance.

## 3 Results

### 3.1 Carbon fiber dust exposure status

Statistical analysis of the job distribution of 955 employees revealed the following: 48 in the storage area, 42 in the mixing area, 460 in the laying area, 144 in the molding area, 160 in the grinding area, and 101 in the material handling area. Based on carbon fiber dust detection results, a total of 80 employees were found to be exposed to carbon fiber dust, accounting for 8.38% of the total; Employees exposed to carbon fiber dust were primarily from the mixing, laying and grinding areas. There were significant differences in the job distribution of employees exposed to carbon fiber dust (*P* < 0.05); however, comparisons of daily working hours, carbon fiber dust test results and test concentrations relative to the OEL showed no statistical significance (*P* > 0.05). See Table 2.

**Table 2:** Exposure to hazardous factors among employees exposed to carbon fiber dust.

Job position	Number of exposed workers	Proportion of personnel in each post (*n*, %)	Daily working hours (h)	Test Result (mg/m^3^)	Detected concentration/OEL
Material storage area	1	2.08 (1/48)	8	1.50	0.50
Molding area	9	6.25 (9/144)	7.55 ± 0.52	1.73 ± 0.10	0.55 ± 0.08
Lay-up area	26	5.65 (26/460)	7.68 ± 3.30	2.28 (2.10, 2.60)	0.75 (0.67, 0.85)
Batching/Mixing Area	13	30.95 (13/42)	7.00 ± 1.84	2.29 (2.20, 2.68)	0.79 (0.70, 0.87)
Polishing/Grinding area	28	17.50 (28/160)	7.50 ± 2.54	2.40 (2.20, 2.70)	0.81 (0.74, 0.90)
Material transfer area	3	1.88 (3/101)	8.00 ± 0.30	1.62 ± 0.09	0.53 ± 0.05
*F/χ^2^*		62.190	1.305	1.135	1.271
*P*		< 0.001	0.295	0.325	0.246

### 3.2 ICMM quantitative assessment results for workers exposed to carbon fiber dust

**Figure 1:**
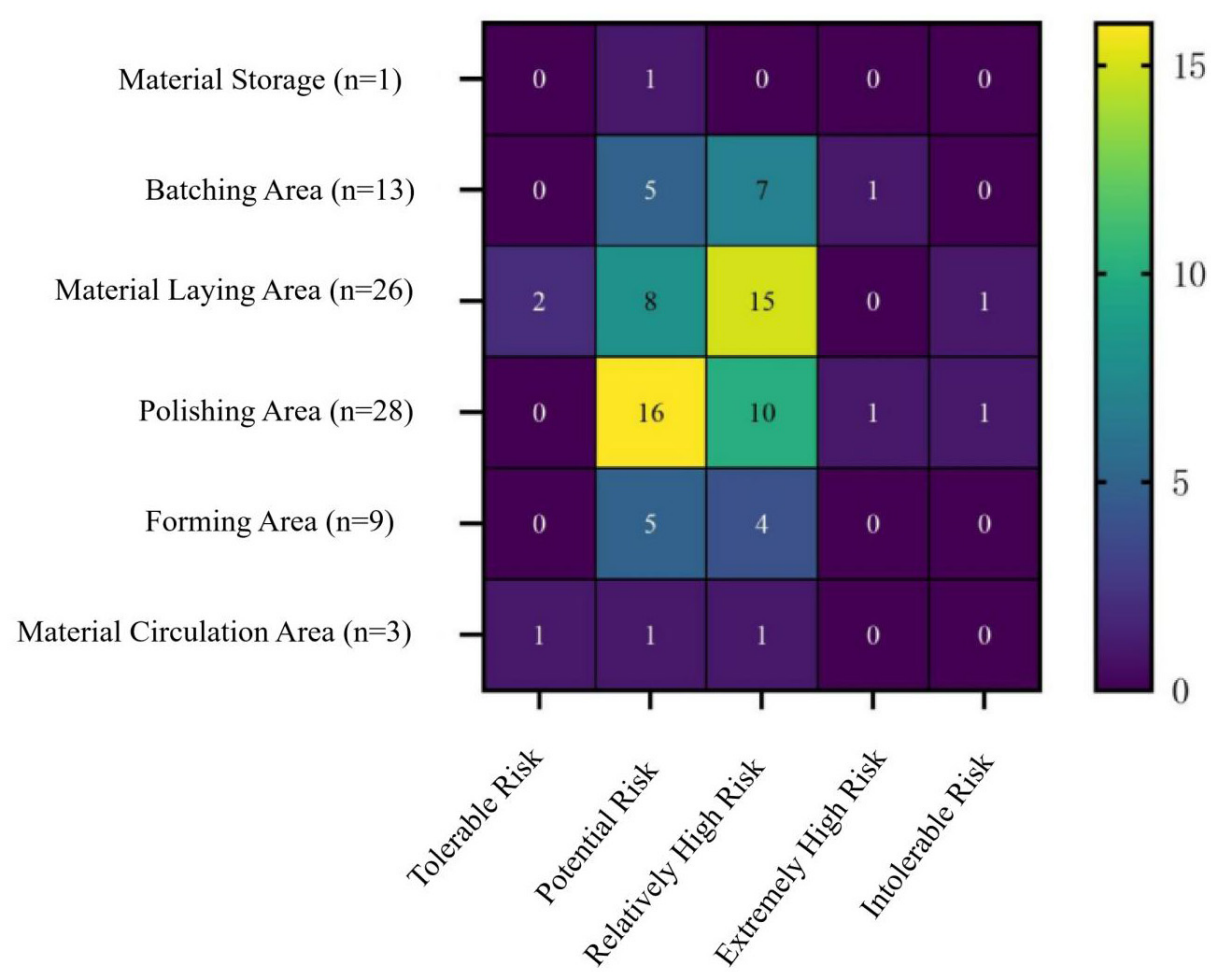
Occupational health risk assessment of employees exposed to carbon fiber dust.

The ICMM model was adopted to assess the occupational health risks of employees exposed to carbon fiber dust. The results showed 3 cases with tolerable occupational health risk, 36 cases with potential occupational health risk, 37 cases with relatively high occupational health risk, 2 cases with extremely high occupational health risk, and 2 cases with intolerable occupational health risk. See Figure 1.

### 3.3 Univariate analysis of carbon fiber dust exposure

The 80 exposed workers were analyzed as a single group, and their ICMM assessment results were compiled. The occupational health risk levels of workers exposed to carbon fiber dust were divided into two categories: the Tolerable + Potential Risk group (*n* = 39) and the High Risk + Very High Risk + Intolerable Risk group (*n* = 41). As shown in Table 3, statistically significant differences (*P* < 0.05) were found between the two groups regarding age, education level, previous years of work in the manufacturing industry, the number of workers in the lay-up area and polishing/grinding area positions, occupational disease prevention awareness, and compliance with personal protective behaviors.

**Table 3:** Univariate analysis of occupational risks among employees in carbon fiber product enterprises.

Variable	Category	High Risk + Very High Risk + Intolerable Risk Group (*n* = 41)	Tolerable + Potential Risk Group (*n* = 39)	χ^2^/t/Z
Age (years)		43.56 ± 6.26	41.00 ± 7.11	1.711, 0.091
Gender	Male	30	29	0.015, 0.904
	Female	11	10	
Education Level	Junior high school, primary school, or illiterate	13	5	10.122, 0.018
	High school, technical secondary school	23	18	
	Associate degree, bachelor's degree	5	15	
	Master's degree or above	0	1	
Years of work in frontline manufacturing positions		7.00 (2.00, 9.00)	3.00 (1.00, 6.00)	2.972, 0.003
Working time in carbon fiber product molding related positions (months)		4.40 ± 1.02	4.28 ± 1.23	0476, 0.635
Job position	Material storage area	0	1	-, 0.488
	Batching/Mixing area	6	7	0.161, 0.688
	Lay-up area	9	17	4.266, 0.039
	Molding area	5	4	0.006, 0.937
	Polishing/Grinding area	20	8	7.020, 0.008
	Material transfer area	1	2	0.002, 0.965
Working days per week (days/week)		6.21 ± 0.75	6.52 ± 0.64	1.984
Occupational disease prevention awareness (score)		60.32 ± 8.65	74.73 ± 8.99	7.307
Personal protective behavior	Compliant	23	31	4.985
	Non-compliant	18	8	
Enterprise protective measures	Effective	27	29	0.689
	Missing or ineffective	14	10	

### 3.4 Variable screening

**Figure 2:**
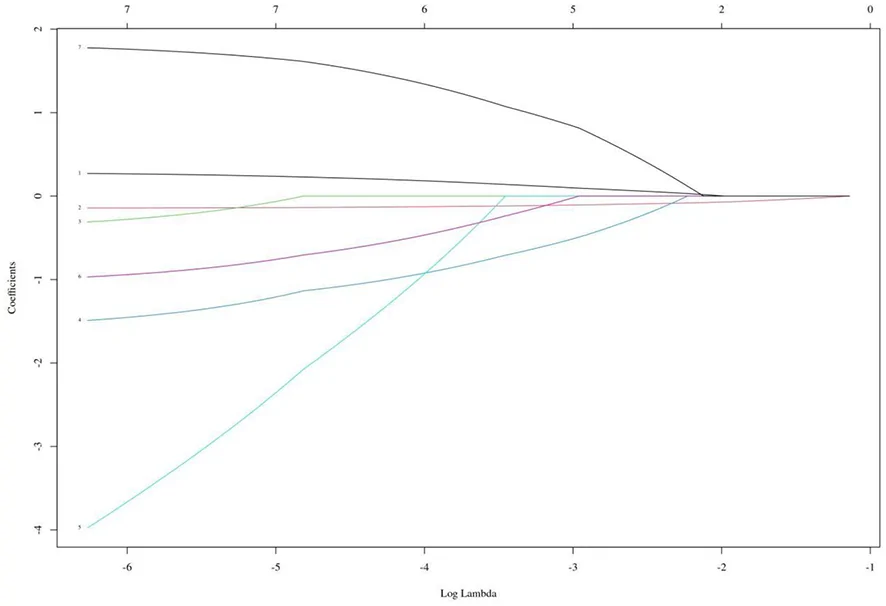
LASSO coefficient path plot.

**Figure 3:**
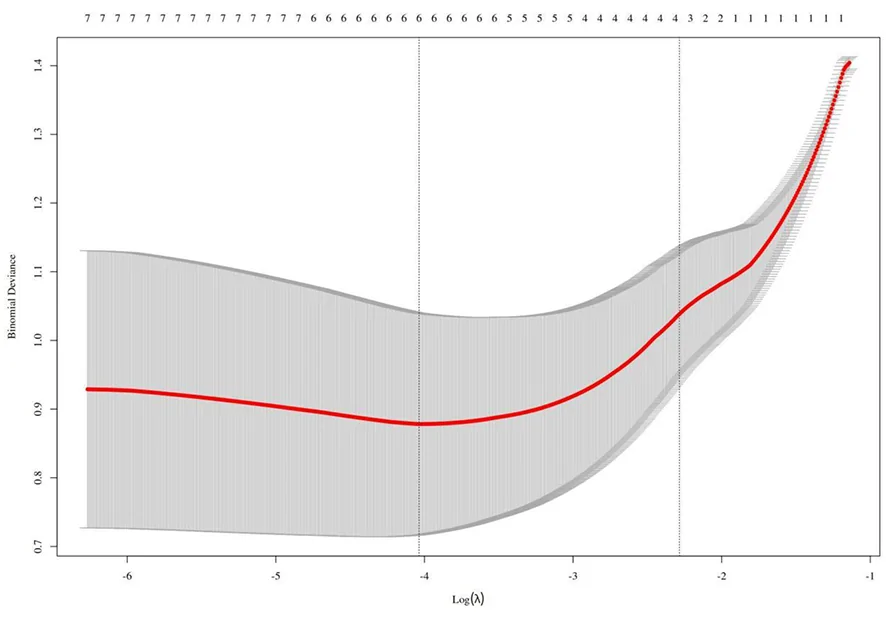
Cross-validation curve.

Given the limited sample size of workers exposed to carbon fiber dust, LASSO regression was performed on indicators with statistically significant differences in univariate analysis to control multicollinearity among variables and improve the robustness of the regression model, with 10-fold cross-validation adopted. The operation results showed that λmin = 0.0176 and λlse = [fill in the value]. Combined with the research objectives, the λmin value was selected for variable penalization. The coefficients of educational level, years of frontline manufacturing work, working in the laying area, working in the polishing area, and awareness of occupational disease protection were all non-zero (Figures 2, 3).

### 3.5 Multivariate analysis of occupational health risks after carbon fiber dust exposure

**Figure 4:**
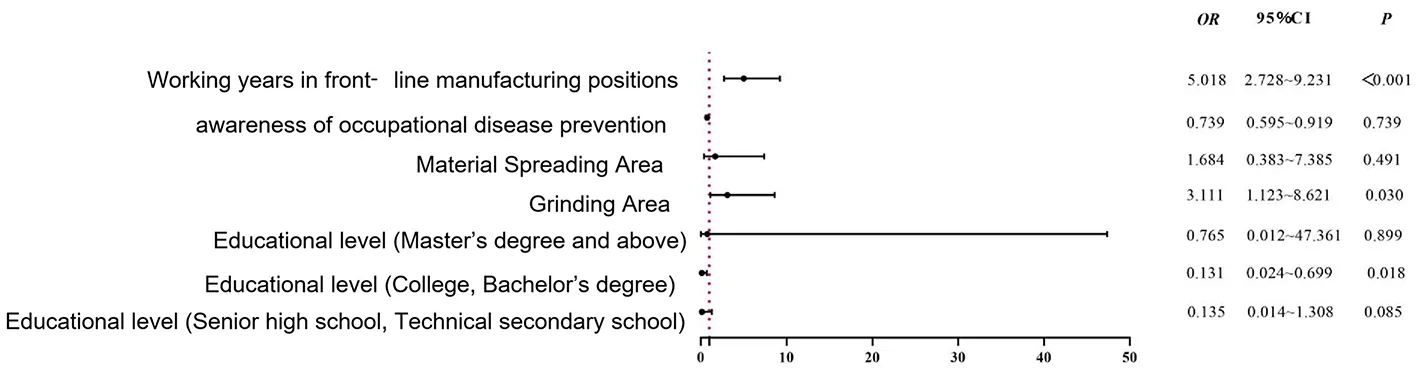
Forest plot of multivariate regression analysis.

Indicators screened by LASSO regression were treated as independent variables, and ICMM occupational health risk was set as the dependent variable. The assignment of each indicator is shown in Table 4. A binary logistic regression model was constructed, and the model fitness test yielded χ^2^=2.361 and *P*=0.967, indicating a good goodness-of-fit of the logistic regression model as shown in Table 5 and Figure 4, variables with no significant difference (*P* > 0.05) were excluded. The working years in front-line manufacturing posts and working in the polishing area were independent risk factors for occupational health risks among employees engaged in carbon fiber product forming. In contrast, higher occupational disease protection awareness and educational level (junior college and bachelor's degree) were protective factors, and all differences were statistically significant (*P* < 0.05).

**Table 4:** Variable assignment for logistic regression.

Type	Specific variable name	Assignment
Dependent variable	Occupational health risk of carbon fiber dust exposure	High Risk + Very High Risk + Intolerable Risk group = 1; Tolerable + Potential Risk group = 0
Independent variable	Education level	Master's degree or above (Z1 = 1, Z2 = 0, Z3 = 0); Associate degree, Bachelor's degree (Z1 = 0, Z2 = 1, Z3 = 0); High school, Technical secondary school (Z1 = 0, Z2 = 0, Z3 = 1); Junior high school, Primary school, or illiterate (Z1 = 0, Z2 = 0, Z3 = 0)
	Years of work in frontline manufacturing positions	Entered as original value
	Job position: Lay-up area	Yes = 1; No = 0
	Job position: Polishing/Grinding area	Yes = 1; No = 0
	Occupational disease prevention awareness	Entered as original value

**Table 5:** Binary logistic regression analysis of occupational health risks among workers exposed to carbon fiber dust.

Variable	β	S.E.	Wald χ^2^	*P*	OR	95% *CI*	
						Lower limit	Upper limit
Years of work in frontline manufacturing positions	1.613	0.311	26.900	< 0.001	5.018	2.728	9.231
Occupational disease prevention awareness	−0.302	0.111	7.402	0.007	0.739	0.595	0.919
Material laying area	0.521	0.755	0.1476	0.491	1.684	0.383	7.385
Polishing/Grinding area	1.135	0.520	4.764	0.030	3.111	1.123	8.621
Education level (Master's degree or above)	−0.268	2.105	0.016	0.899	0.765	0.012	47.361
Education level (Associate degree, Bachelor's degree)	−2.036	0.856	5.657	0.018	0.131	0.024	0.699
Education level (High school, Technical secondary school)	−2.001	1.58	2.986	0.085	0.135	0.014	1.308
Constant	4.356	1.522	8.191	0.005	77.945	–	–

## 4 Discussion

Although some studies suggest that industrial carbon fibers typically have a large diameter, making it difficult for them to penetrate deep into the lungs, and that carbon fiber is chemically stable and generally does not react within biological organisms, processes such as grinding and machining of carbon fiber materials can generate carbon fiber dust with an aerodynamic equivalent diameter of 0.4 μm. This is particularly significant during operations like filament winding. Carbon fiber dust exposure is an issue that the carbon fiber product molding industry must confront (14). This type of fibrous dust can enter the deep lungs through respiration. If combined with nanoscale fibrous dust, the exposure hazard is further amplified. Additionally, carbon fiber dust is hard and sharply shaped, which can cause physical skin damage and can also enter the eyes and bloodstream, posing potential long-term risks to employees (15). Carbon fiber dust is a major concern for personnel in the carbon fiber molding industry. Investigating the occupational health risks and related factors for this exposed population is of great significance for the health management of these workers. There are discrepancies in the control stringency, policies and regulations, sampling methods, and limit criteria for carbon fiber dust across different countries. The revised Chinese national standard GBZ 2.1-2019 has deleted the independent limit item for carbon fiber dust adopted in previous editions and classifies carbon fiber dust under “other dust” for mandatory limit management. The present study adopted the mandatory standard for respirable dust with the mass concentration method for calculation. By contrast, the National Institute for Occupational Safety and Health (NIOSH) of the United States and Japan apply the Recommended Exposure Limit (REL) and assess the REL level of inhalable fibers using the fiber counting method. Significant errors may occur after mandatory conversion due to the fundamental differences between the two measurement approaches. Affected by the wide variation in the aspect ratio and diameter of carbon fiber debris, the fiber counting method yields far lower results than the mass concentration method under the same working scenario, indicating that the foreign control criteria are stricter than domestic standards.

A total of 955 employees involved in this study were engaged in six front-line posts during the forming process of carbon fiber products. The proportion of workers in the material laying area was the highest at 48.17%, followed by the polishing area (16.75%), while the batching area accounted for the lowest proportion (4.40%). Combined with the production characteristics of carbon fiber products, the differences in post proportion are mainly attributed to variations in job types and equipment automation levels.

In the present statistical results, 8.38% of employees were exposed to carbon fiber dust. Although no horizontal comparative data are available, the findings suggest that occupational disease prevention and control measures should be further strengthened in carbon fiber product molding enterprises in Shenzhen. Hazard exposure does not necessarily lead to the occurrence of occupational diseases. Therefore, this study adopted the ICMM model to explore the occupational health risk levels of employees exposed to carbon fiber dust. The ICMM model comprises two evaluation approaches: quantitative assessment and matrix qualitative assessment. Previous studies have widely applied the ICMM model to evaluate occupational health risks in workplace settings (16, 17). For instance, Ji Jingyun et al. (18) conducted both quantitative and qualitative ICMM assessments of occupational exposure risks across different posts, and their results indicated that only winding workers had carbon fiber dust exposure risk, which was classified as tolerable.

In accordance with the research objectives, only the quantitative assessment module of the ICMM model was adopted in this study. The results showed an overall high level of occupational health risk, with most participants distributed in the potential and relatively high-risk categories. Such discrepancies in risk grading are mainly attributed to two factors. First, significant differences in study samples. The PrE scores in the present study were generally high (6 or 10 points). Combined with the standard 8-h working system and frequent overtime work in manufacturing workshops, the actual dust exposure duration and exposure intensity were further increased, which substantially elevated the final comprehensive risk scores. Second, the original ICMM scoring system adopts discrete grade jumping assignment for the C value. Under the multiplicative exponential amplification effect inherent in the ICMM model, minor differences in individual indicators can lead to marked enlargement of the final risk grade.

In addition, slight variations in PrE scores were observed among employees in the same post, which can be explained by individual operational habits (e.g., operating distance and posture), diverse working modes (e.g., rough grinding, precision grinding, and trimming), and different standardization levels of personal protective behaviors. In the analysis of occupational health risk levels among employees exposed to carbon fiber dust, in view of the small sample size of exposed subjects, numerous candidate variables and the demand for model robustness, LASSO regression was further used to eliminate variables after preliminary univariate screening. This approach can identify multicollinear variables to improve model robustness, prevent model overfitting, and enhance the generalization performance of the model. The results of binary Logistic regression were as follows: (1) Years of work in frontline manufacturing positions: There was a positive relationship between years of work in frontline positions and the occupational health risk of carbon fiber dust in carbon fiber product molding positions. This was the third largest influencing factor in this study. Certain positions in manufacturing share common characteristics, such as dust, noise, and mechanical hazards. The longer an employee has worked in previous frontline positions, the greater the accumulated exposure damage may be (19, 20). Although these may not be carbon fiber dust exposure positions, the body's self-repair capacity for occupational injury weakens with prolonged exposure time. Upon entering the carbon fiber product molding industry, the body's ability to resist and repair damage decreases, lowering the threshold for harm from carbon fiber dust. Under equivalent exposure levels, such employees are more prone to occupational diseases (21–23). Furthermore, the longer an employee works in similar positions, the more obvious occupational protection vulnerabilities may become due to habituation, empiricism, and complacency. (2) Occupational disease prevention awareness and personal protective behavior: For positions with inevitable occupational health exposure risks, personal protective awareness and behavior are key factors determining the final exposure outcome. They serve as the first line of defense against occupational exposure and disease (24) and directly determine the level of exposure risk. This survey found that although some employees clearly understood the consequences of occupational exposure, they harbored (a sense of invincibility or taking chances) in their daily work and focused more on “immediate harm.” Employees had higher vigilance toward positions with clear risks and immediate injuries. For example, the work characteristics of the polishing/grinding area and batching/mixing area determine the high risk of carbon fiber dust exposure and related occupational diseases for employees in these positions; therefore, these employees performed better self-protection. In contrast, employees in positions like the lay-up area and material storage area, where typical occupational hazards are work-related musculoskeletal disorders or VOCs, paid less attention to carbon fiber dust exposure, making them more prone to protection gaps. (3) Polishing/Grinding area positions: Employees exposed to carbon fiber dust are mainly stationed in the batching area, laying area and polishing area, which is consistent with the production characteristics of these posts. The polishing workshop generates the highest dust concentration throughout the carbon fiber molding process, with the greatest difficulty in hazard control and the longest daily exposure duration to harmful agents for workers. During dry high-speed grinding operations, cured carbon fiber-reinforced polymer composites are mechanically abraded, breaking the resin matrix and carbon fiber structures and releasing massive amounts of free carbon fiber dust. In manual polishing operations, workers' mouths and noses stay very close to the dust-emitting points of grinding tools, with insufficient space for dust dispersion and sedimentation. In addition, polishing longitudinally splits carbon fibers into respirable fibrous dust with diameters of 2–5 μm. These tiny fibers remain suspended in the air for a long time and can easily penetrate ordinary dust masks. Combined with the irreversible respiratory damage induced by carbon fibers, multi-path exposure pathways and latent clinical symptoms, workers in this post ultimately face elevated occupational health risks. (4) Education level: In this study, employees with higher education levels had lower assessed occupational health risks. This may be related to higher levels of personal protection awareness among more educated employees. Such employees do not completely prioritize performance above all else. Furthermore, some enterprises include safety incidents as one of the evaluation criteria for job promotion. Higher-educated employees, having more promotion potential, may pay more attention to controlling safety incidents.

In conclusion, the prevalence of carbon fiber dust exposure among employees in this study was 8.38%. ICMM model evaluation indicated that the occupational health risks of exposed workers were predominantly at the potential and relatively high levels. Working years in front-line manufacturing posts and workplace types were identified as independent risk factors for elevated occupational health risks, while educational level and occupational disease protection awareness also significantly correlated with individual risk levels. These findings suggest that enterprises can carry out targeted safety optimization and production reform based on the above risk factors. Specific measures include incorporating air quality monitoring of non-dust working areas into daily safety management, so as to strengthen occupational health protection and promote the sustainable and safe development of the carbon fiber product forming industry. However, all participants in this study were recruited from enterprises in Shenzhen, which may limit the generalizability and extrapolation of the findings. Future research can expand the sampling scope to improve the practical application value of the established model. In addition, this study adopted a cross-sectional design, which cannot clarify the temporal sequence between variables and outcomes, and the risk of reverse causality cannot be ruled out.

## Data Availability

The raw data supporting the conclusions of this article will be made available by the authors, without undue reservation.
